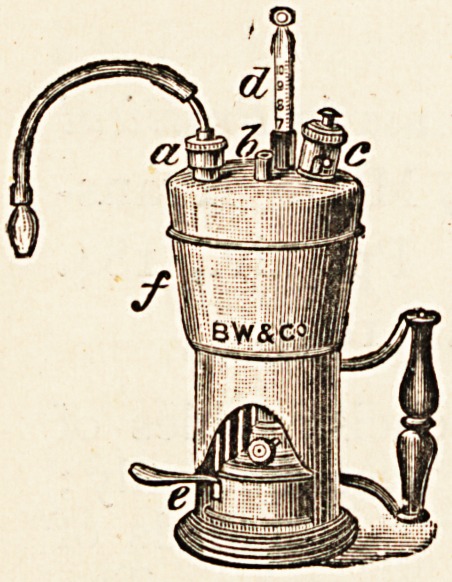# Notes on Preparations for the Sick

**Published:** 1892-09

**Authors:** 


### Notes on Preparations for the Sick.

**The Hot Air Inhaler.**—Burroughs, Wellcome & Co., London.

Explanation.—*a* Box with sponge for inhalants. *b* Inlet for air. *c* Safety valve. *d* Thermometer. *e* Spirit lamp. *f* Boiler.

This inhaler, suggested by the late Sir Morell Mackenzie, is considered to be an improvement on the idea of Weigert. The boiler may be partly filled with either water, glycerine, or oil, according to the temperature desired for the air to be inhaled. The box *a* (shown on the sketch) contains a sponge which may be saturated with any volatile medicament; then, as the hot air is drawn through the aperture *b* and makes its exit from the mouthpiece, the inhalant is volatilized and conveyed into the lungs and air passages. The boiler contains a coil of metal tubing, and the air drawn through it becomes thoroughly heated to the temperature of the boiling liquid. This boiler is made of very strong material, and is fitted with a safety valve, so that there is very little chance of an accident occurring. The lamp may be filled with methylated spirit, and contains sufficient spirit to heat to boiling point the boiler when half full of glycerine. Full directions for use are furnished with the instrument.

Although the inhalation of hot air in phthisis has been now proved to be useless as an anti-bacillary agent, yet the therapeutic uses of hot air in catarrhal conditions of the tubes are likely to be abundantly demonstrated by the introduction of so efficient an inhaler as this.

---

**Pepsalia.**—G. & G. Stern, London.

This digestive table salt is so well known that it needs no description. It certainly has considerable digestive power, and is a useful adjunct to the food in cases where the digestion needs a little artificial assistance.

The mode of administration ensures a thorough admixture with the food, and enables the minimum amount of pepsine to do the maximum amount of work.

**Surgeon's Silk Gelatole Plaster. Rubber Adhesive Plaster. Belladonna Plaster. Capsicum Plaster. Canthos, or Cantharidal Plaster. Prepared Linseed Poultice.**—JOHNSON AND JOHNSON, LIMITED, London.

These plasters are all of the most elegant and convenient character, and are truly useful both to surgeon and patient.

The **Silk Gelatole Plaster** is intended to take the place of isinglass plaster, and is applied by moistening the adhesive side. It is very thin and of a pale pink colour, so that when applied to a cut finger on a lady's delicate hand its presence can hardly be detected. The **Rubber Adhesive Plaster** is now found in every well-furnished surgery. It adheres so closely and evenly to the skin, and holds so tenaciously after application, that it creates a new era in the use of strapping plaster, while its waterproof nature renders it very little liable to become loosened when applied in situations which are constantly moist. We have found it very useful after operations for hare-lip. The **Belladonna Plaster** is prepared in combination with boracic acid, which causes an increased action and more immediate effect. It is prepared both perforated and plain, and in the latter form we have found it very comfortable and efficacious. The **Capsicum Plaster, Cantharidal Plaster,** and **Prepared Linseed Poultices** are all handy preparations designed to save time and trouble in cases of emergency, and may be relied on to produce the desired effects, with a minimum of discomfort to the patient.

## Figures and Tables

**Figure f1:**